# Proteomic identification of moesin upon exposure to acrolein

**DOI:** 10.1186/s12953-017-0130-4

**Published:** 2018-01-17

**Authors:** Pureun-Haneul Lee, Byeong-Gon Kim, Sun-Hye Lee, George D. Leikauf, An-Soo Jang

**Affiliations:** 1 Division of Allergy and Respiratory Medicine, Department of Internal Medicine, Soonchunhyang University Bucheon Hospital, 170 Jomaru-ro, Wonmi-gu, Bucheon, Gyeonggi-do 420-767 South Korea; 2 Department of Environmental and Occupational Health, Graduate School of Public Health, University of Pittsburgh, Pittsburgh, PA USA

**Keywords:** Acrolein, Asthma, HMVEC-L, Moesin, Proteomics

## Abstract

**Background:**

Acrolein (allyl Aldehyde) as one of smoke irritant exacerbates chronic airway diseases and increased in sputum of patients with asthma and chronic obstructive lung disease. But underlying mechanism remains unresolved. The aim of study was to identify protein expression in human lung microvascular endothelial cells (HMVEC-L) exposed to acrolein.

**Methods:**

A proteomic approach was used to determine the different expression of proteins at 8 h and 24 h after treatment of acrolein 30 nM and 300 nM to HMVEC-L. Treatment of HMVEC-L with acrolein 30 nM and 300 nM altered 21 protein spots on the two-dimensional gel, and these were then analyzed by MALDI-TOF MS.

**Results:**

These proteins included antioxidant, signal transduction, cytoskeleton, protein transduction, catalytic reduction. The proteins were classified into four groups according to the time course of their expression patterns such as continually increasing, transient increasing, transient decreasing, and continually decreasing. For validation immunohistochemical staining and Western blotting was performed on lung tissues from acrolein exposed mice. Moesin was expressed in endothelium, epithelium, and inflammatory cells and increased in lung tissues of acrolein exposed mice compared with sham treated mice.

**Conclusions:**

These results indicate that some of proteins may be an important role for airway disease exacerbation caused by acrolein exposure.

**Electronic supplementary material:**

The online version of this article (10.1186/s12953-017-0130-4) contains supplementary material, which is available to authorized users.

## Background

Smoking is a major risk factor for morbidity and mortality worldwide [1]. Cigarette smoking and asthma are associated with poor symptom control and impaired therapeutic responses to antiasthma drugs [2, 3]. Compared with asthmatic nonsmokers, smokers with asthma have worse symptom control [4], an accelerated decline in lung function [5], and an increased mortality rate [6]. Steroid treatment and smoking cessation can improve symptom and lung function of asthma [7, 8]. Increased number, size and density of blood vessels, as well as vascular leakage and plasma engorgement, have been reported in the airways of patients with all grades of asthma from mild to fatal [9].

Angiogenesis relies on the proliferation and migration of endothelial cells [10]. The importance of endothelial cell proliferation for expansive growth of the vascular network has long been recognized [11]. The endothelium is a monolayer of cells lining the interior of the blood and lymphatic vessels. This cellular layer is attached to the basal membrane and participates in the exchange of materials between blood and tissues. Endothelial cells have essential activities in the control of vascular functions and play an important role in the formation of new blood vessels and restoration of damaged vessels [12, 13]. Endothelial cells release a multitude of biological mediators such as growth factors, vasoactive mediators, coagulation and fibrinolysis proteins, and immune factors. These cells are usually in the quiescent state, reflecting the stability and integrity of the vascular wall [14–18].

Acrolein (2-propenal) is an α, β-unsaturated aldehyde that is volatile at room temperature and is highly irritating to eyes and respiratory passages [19]. Acrolein can be formed by heating cooking oils and fats above 300 °C [20], and can be formed in domestic cooking with biomass fuels [21], and is present in environmental tobacco smoke [19, 22], which remains a significant occupational health hazard in the restaurant workplace [23, 24]. Acrolein levels were higher in induced sputum and in exhaled breath condensate in both asthma and COPD [25]. Acrolein induces changes in a diverse range of proteins that may be related to cellular toxicity in rat lung epithelial cells and that toxic effects of acrolein may be due to deregulation of proteins involved in proliferation and apoptosis [26], and has the potential to modify a wide variety of cysteine-containing proteins within airway epithelial cells [27].

By proteomic approach, we have been able to address whether timing and dose exposures of acrolein influence endothelial protein expression. Two dimentional electroporesis data was validated in acrolein exposed mice.

## Methods

### Cell culture and stimulation with Acrolein

Primary human lung micro vascular endothelial (HMVEC-L) cells (Lonza, Switzerland) (5000cells/cm^2^) were grown in EGM™-2MV BulletKit™ (Lonza, Basel, Switzerland). The medium was replaced every 48 h until cells reached 90% confluence at 37 °C under 5% CO_2_. The cells are seeding in 150cm^2^ plate. For experimental treatment, HMVEC-L were re-cultured in EGM™-2MV with 0.1% FBS for 30 min, and Treatment of the various concentrations of Acrolein cultured at 37 °C under 5% CO2 incubator for 8 h and 24 h.

### Two-dimensional (2-D) electrophoresis and image analysis

HMVEC-L cells were harvested by centrifugation and then disrupted with lysis buffer containing 5 mM Tris-HCl (pH 7.4), 100 mM NaCl, 1% Triton X-100, and 2 mM PMSF. The cell lysate was centrifuged at 12,000 Χ *g* for 30 min, and the supernatant fraction was collected. Protein concentrations were determined using a BCA assay kit (Pierce). Immobiline DryStrips (Amersham Biosciences) were used for isoelectric focusing, which was carried out with 1 mg of the extracted protein on an IPGphor system (Amersham Biosciences). After IEF separation, the proteins were separated in the second dimension by SDS-PAGE. For image analysis, the gels were visualized with Coomassie Brilliant Blue G-250 according to the manufacturer’s instructions. The 2-D gels were scanned with an ImageScanner (Amersham Biosciences) in transmission mode. Spot detection and matching were performed using ImageMaster 2D version 5.0 (Amersham Biosciences). Digitized images were analyzed using the ImageMaster program to calculate the 2-D spot intensity by integrating the optical density over the spot area (the spot “volume”) and normalized. The values were normalized and then exported to SPSS 18.0 for statistical analysis.

### Protein identification by MALDI-TOF MS analysis and database searching

Mass spectrometry (MS) was performed at the Yonsei Proteome Research Center (YPRC, Korea). Peptides obtained after trypsin digestion from cell lysate were analyzed with a MALDI-TOF/TOF mass spectrometer (4800 ABSciex, USA). For sample preparation, 70% ACN was allowed to flow through the porous tip to remove impurities attached to the resin. Next, a solution of 2% formaldehyde was passed through the column to create acidic conditions. Following this pretreatment, the samples to be analyzed were passed through the column. Subsequently, the wash buffer (2% FA) was used to remove impurities such as salt and chemicals. Finally, the peptides attached to the resin were elute.

### Database search and protein identification

MS and MS/MS data were used in subsequent searches by Mascot software (Matrix Science; http://www.matrixscience.com) using the MSDB protein sequence database for human proteins.

### Acrolein exposure and histology examination

This study was performed in accordance with the Institutional Animal Care and Use Committee of the Soonchunhang University Bucheon Hospital and mice were housed under specific pathogen free conditions. 6- to 8-week-old BALB/c female mice (*n* = 8 mice; Orient Bio, Sungnam, Korea) were used in the study. Animals were exposed to filtered air (control) or Ovalbumin (3μg/ul) or acrolein (5 ppm, 6 h) generated and monitored as described previously [28].

To examine acrolein-induced changes in lung histology, Mice were anesthetized by intraperitoneal injection of with 2.5 mg/kg tiletamine and xylazine (Zoletil and lumpum; Bayer Korea Co., Seoul, Korea). The following day BALF was obtained, centrifuged, and the supernatant stored (-20 °C). The cell pellet was resuspended for cell count and cytospin slides were made for cell differential (500 cells/mouse, Diff-Quick-staining). A portion of the lung was fixed in 4% phosphate-buffered paraformaldehyde, embedded in paraffin, sectioned, and stained.

Fixed lungs were washed with Dulbecco’s Phosphate-Buffered Saline containing Ca2+, Mg2+, 6.1 mM D-glucose, and 0.33 mM sodium pyruvate (DPBS; Cat. No. 14287–080, Life Technologies, Carlsbad, CA), dehydrated through a series of graded ethanol solutions (30–70%), and processed in paraffin blocks (Hypercenter XP, Shandon, Ramsey, MN). The lung tissue was sectioned (4 μm) and stained with hematoxylin and eosin. Total protein in cell-free supernatants was measured using a bicinchoninic acid assay (BCA; Thermo Scientific, USA) using bovine serum albumin as a standard.

### Determination of airway responsiveness to methacholine

Airway responsiveness was measured in unrestrained, conscious mice 1 day after the last challenge, as described previously [29]. Mice were placed in a barometric plethysmographic chamber (All Medicus Co, Anyang, Korea) and baseline readings were taken and averaged for 3 min. Increasing concentrations of aerosolized methacholine, from 0 to 100 mg/mL, were nebulized through an inlet of the main chamber for 3 min. Readings were taken and averaged for 3 min after each nebulization, and the enhanced pause (Penh) was determined. Penh, calculated as (expiratory time/relaxation time-1) Χ (peak expiratory flow/peak inspiratory flow), is a dimensionless value that represents a function of the proportion of maximal expiratory to maximal inspiratory box pressure signals and is a function of the timing of expiration. Penh was used as a measure of airway responsiveness to methacholine. Results were expressed as the percentage increase in Penh following challenge with each concentration of methacholine, where the baseline Penh (after saline challenge) was expressed as an actual value. Penh values averaged for 3 min after each nebulization were evaluated.

### Western blot analysis of Moesin expression

The extracted lung tissues were homogenized in a protein lysis solution containing 50 mM Tris-HCL (pH 7.4), 50 mM NaCl, 0.1% SDS, 1% Triton X-100, 0.5 mM EDTA, and 100 mM PMSF in distilled water, and centrifuged at 14000 rpm for 30 min at 4 °C, and the insoluble materials were collected. Proteins were fractionated by 10% SDS-PAGE and transferred to a PVDF membrane (millipore). The membrane was blocked for 1 h in TBS containing 5% BSA and then incubated with polyclonal rabbit anti-Moesin antibody (1:1000 dilution) at 4 °C overnight. The unbound primary antibodies were removed with three 10-min washes in TBS containing 0.01 Tween-20. The membrane was then incubated with goat anti-rabbit for 1 h. ECL detection of moesin was performed according to the manufacturer’s instructions (Roche Applied Science).

### Analysis of Moesin expression in lung tissue of Acrolein exposured mouse by Immunohistochemical staining

Mouse lung sections were deparaffinizied and rehydrated in an ethanol series to examine the secretion of Moesin. Four-micrometer tissue section slides were treated with 0.3% H_2_O_2_-methanol for 30 min to block endogenous peroxidase and then incubated with Moesin antibody (1:100 dilution, abcam) at 4 °C overnight. After washing with Tris-buffered saline the slides were incubated with avidin-biotin peroxidase complex (ABC kit, Vector Laboratory, Burlingame, CA). Color reaction was developed by staining with liquid DAB+ substrate kit (Golden Bridge International Inc., Mukilteo, WA). After immunohistochemical staining the slides were counterstaining the slide with Herris’s hematoxylin for 1 min.

### Statistical analysis

Statistical analysis was performed using SPSS 18.0 software. Differences in spot intensity on 2-D gels were multiple compared between three independent groups or samples using the non-parametric Kruskal-Wallis *H* test for continuous data. If differences were found significant, the Mann-Whitney *U* test (two-sample rank sum test) was applied to the differences in densities and concentrations of the groups. All data were expressed as median values (interquartile range), and significance was defined as *p <* 0.05.

## Results

### 2-D electrophoresis and protein identification

**Fig. 1:**
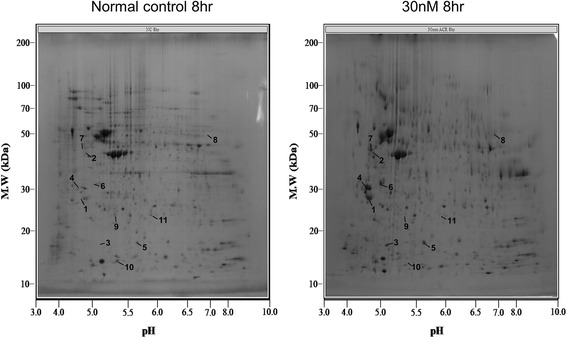
Two-dimensional electrophoresis of human lung microvascular endothelial cells. The 2-D PAGE image from lysates of untreated cells was used as a master gel and reference map. Acrolein 30 nm treatment caused 11 spots to change by more than 3-fold at 8 or 24 h. Protein spots identified by MALDI-TOF MS (arrow) are marked by their *spot numbers*

**Fig. 2:**
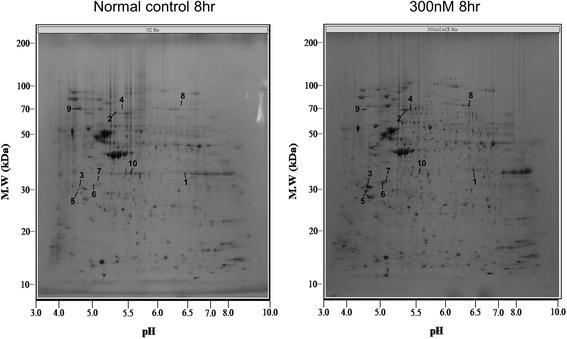
Two-dimensional electrophoresis of human lung microvascular endothelial cells.The 2-D PAGE image from lysates of untreated cells was used as a master gel and reference map. Acrolein 300 nm treatment caused 10 spots to change by more than 3-fold at 8 or 24 h. Protein spots identified by MALDI-TOF MS (arrow) are marked by their *spot numbers*

A proteomics approach was used to determine the differential expression of proteins at 8 and 24 h after treatment of HMVEC-L cells with 30 nM and 300 nM acrolein. Protein fraction were obtained by differential centrifugation and then separated by 2-D electrophoresis in six replicate gels per treatment. A representative image of the proteomic profile of HMVEC-L cells prior to treatment is shown in Figs. 1 and 2. A total of 427 (median, 425; range, 400–460) protein spots were detected on each gel. All of the identified spots were localized in the pI3–10 range with a molecular mass range of 10–200 kDa. This 2-D gel image was used as a master gel and reference map. 2-D PAGE of extracts from acrolein-treated cells revealed 11 spots for 30 nM and 10 spots for 300 nM that changed by more than 3-fold at 8 or 24 h after treatment. These spots were excised from the gel and incubated with trypsin to digest the proteins in the gel, which were then analyzed by MALDI-TOF MS. The results of this analysis are summarized in Table 1, 2, 3 and 4.

**Table 1:** List of proteins identified by MALDI-TOF MS analysis – 30 nM 8 h

No.	Protein name	Abbreviation	Accession no.	Amino acid sequence	pI/molecular mass (Da)	Normalized relative intensity			Ratio	*p*-value	Function
						Untreat		Treat(8 hr)			
Group 1											
1	adenylyl cyclase-associated protein	CAP	178,084	R.SALFAQINQGESITHALK.H	51.926/8.07	0.112379	<	0.606937	5.4008	0.001088	Catalyzing reduction
2	Annexin A5	ANXA5	113,960	K.GLGTDEESILTLLTSR.S	35.971/4.94	0.147797	<	1.02077	6.90659	0.000050	Signal transduction
3	14–3-3 protein epsilon isoform	YWHAE	902,787	K.VAGMDVELTVEER.N	29.326/4.63	0.176267	<	1.13002	6.41086	0.003154	Signal transduction
4	phospholipase A2	PLA2	189,953	K.FLIPNASQAESK.V	27.899/4.73	0.43137	<	1.47095	3.40996	0.000393	Protein modification
5	initiation factor 4D	eIF-4D	181,997	K.VHLVGIDIFTGK.K	17.049/5.08	0.225855	<	0.92464	4.09396	2.5716E-8	Signal transduction
6	fibrin beta		223,002	K.HQLYIDETVNSNIPTNLR.V	51.358/7.95	0.167372	<	1.21898	7.28303	0.001577	Coagulation
Group2											
7	Tropomyosin beta chain	TPM2	136,090	R.KLVILEGELER.S	32.945/4.66	0.202888	<	0.929909	4.58336	0.000002	Cytoskeleton
8	vimentin	VIM	340,219	R.ETNLDSLPLVDTHSKR.T	53.738/5.03	0.0930222	<	0.363739	3.91024	0.000004	Cytoskeleton
Group 3											
9	glyceraldehyde-3-phosphate dehydrogenase	GAPDH	31,645	K.LISWYDNEFGYSNR.V	36.202/8.26	0.712529	>	0.0703642	0.09875	0.000019	Catalyzing reduction
10	Glutathione S-transferase P	GST-π	121,746	R.TLGLYGKDQQEAALVD MVNDGVEDLR.C	23.569/5.43	0.371254	>	0.0795455	0.21426	0.000011	Antioxidation
Group 4											
11	Glutathione S-transferase P	GST-π	121,746	R.TLGLYGKDQQEAALVD MVNDGVEDLR.C	23.569/5.43	0.342932	>	0.102187	0.29798	0.000009	Antioxidation

**Table 2:** List of proteins identified by MALDI-TOF MS analysis – 30 nM 24 h

No.	Protein name	Abbreviation	Accession no.	Amino acid sequence	pI/molecular mass (Da)	Normalized relative intensity			Ratio	*p*-value	Function
						Untreat		Treat(24 h)			
Group 1											
1	adenylyl cyclase-associated protein	CAP	178,084	R.SALFAQINQGESITHALK.H	51.926/8.07	0.155175	<	0.255949	1.64942	0.000459	Catalyzing reduction
2	Annexin A5	ANXA5	113,960	K.GLGTDEESILTLLTSR.S	35.971/4.94	0.215959	<	0.530059	2.45445	0.000099	Signal transduction
3	14–3-3 protein epsilon isoform	YWHAE	902,787	K.VAGMDVELTVEER.N	29.326/4.63	0.257559	<	0.601467	2.33526	6.5816E-9	Signal transduction
4	phospholipase A2	PLA2	189,953	K.FLIPNASQAESK.V	27.899/4.73	0.363461	<	0.822213	2.26218	0.000102	Protein modification
5	initiation factor 4D	eIF-4D	181,997	K.VHLVGIDIFTGK.K	17.049/5.08	0.309087	<	0.467838	1.51361	0.000093	Signal transduction
6	fibrin beta		223,002	K.HQLYIDETVNSNIPTNLR.V	51.358/7.95	0.594501	<	0.372705	1.59509	0.000108	Coagulation
Group 2											
7	Tropomyosin beta chain	TPM2	136,090	R.KLVILEGELER.S	32.945/4.66	0.296457	<	1.09976	3.70968	5.8027E-7	Cytoskeleton
8	vimentin	VIM	340,219	R.ETNLDSLPLVDTHSKR.T	53.738/5.03	0.159818	<	0.858538	5.37197	0.000118	Cytoskeleton
Group 3											
9	glyceraldehyde-3-phosphate dehydrogenase	GAPDH	31,645	K.LISWYDNEFGYSNR.V	36.202/8.26	0.835329	>	0.395561	0.47354	0.000003	Catalyzing reduction
10	Glutathione S-transferase P	GST-π	121,746	R.TLGLYGKDQQEAALVD MVNDGVEDLR.C	23.569/5.43	0.40715	>	0.149978	0.36836	0.000013	Antioxidation
Group 4											
11	Glutathione S-transferase P	GST-π	121,746	R.TLGLYGKDQQEAALVD MVNDGVEDLR.C	23.569/5.43	0.296094	>	0.093522	0.31585	0.000076	Antioxidation

**Table 3:** List of proteins identified by MALDI-TOF MS analysis – 300 nM 8 h

No.	Protein name	Abbreviation	Accession no.	Amino acid sequence	pI/molecular mass (Da)	Normalized relative intensity			Ratio	*p*-value	Function
						Untreat		Treat(8 h)			
Group 1											
1	Annexin A1	ANXA1	34,388	K.GVDEATIIDILTKR.N	6.57 /38918	0.06388	<	0.34043	5.32922	0.000218	Signal transduction
2	71 Kd heat shock cognate protein	HSP7C	32,467	K.SFYPEEVSSMVLTK.M	5.37 /71082	0.10343	<	0.525932	5.08493	0.000253	Protein translocation
3	Tropomyosin 4	TPM4	12,803,959	R.KIQALQQQADEAEDR AQGLQR.E	4.67/28667	0.15968	<	0.785374	4.91843	0.000554	Cytoskeleton
4	71 Kd heat shock cognate protein	HSP7C	32,467	K.SFYPEEVSSMVLTK.M	5.37/71082	0.109243	<	0.455133	4.16623	1.1276E-7	Protein translocation
5	14–3-3 protein epsilon isoform	YWHAE	902,787	R.VTIASLPR.N	4.63/29326	0.138728	<	0.536942	3.87054	0.000502	Signal transduction
6	Annexin A5	ANXA5	37,637	R.YLAEFATGNDR.K	4.94/35971	0.06177	<	0.228443	3.6983	8.0228E-7	Signal transduction
7	Annexin A5	ANXA5	37,637	R.LYDAYELK.H	4.94/35971	0.116321	<	0.325831	2.80113	0.000015	Signal transduction
Group 2											
8	moesin, isoform CRA_b		119,625,804	K.GLGTDEESILTLLTSR.S	5.90 /66678	0.184177	<	0.519492	2.82061	0.000595	Cytoskeleton
Group 3											
9	Glucose-regulated protein 78 precursor, partial	GRP78	386,758	R.TWNDPSVQQDIK.F	5.03 /72185	0.803415	>	0.232592	0.28950	0.002023	Signal transduction
Group 4											
10	glyceraldehyde-3-phosphate dehydrogenase	GAPDH	31,645	R.GALQNIIPASTGAAK.A	8.26 /36202	0.560738	>	0.203281	0.36252	0.000884	Catalyzing reduction

**Table 4:** List of proteins identified by MALDI-TOF MS analysis – 300 nM 24 h

No.	Protein name	Abbreviation	Accession no.	Amino acid sequence	pI/molecular mass (Da)	Normalized relative intensity			Ratio	*p*-value	Function
						Untreat		Treat(24 h)			
Group 1											
1	Annexin A1	ANXA1	34,388	K.GVDEATIIDILTKR.N	6.57 /38918	0.06388	<	0.230679	3.61114	0.000003	Signal transduction
2	71 Kd heat shock cognate protein	HSP7C	32,467	K.SFYPEEVSSMVLTK.M	5.37 /71082	0.10343	<	0.449127	4.34234	4.8387E-7	Protein translocation
3	Tropomyosin 4	TPM4	12,803,959	R.KIQALQQQADEAEDR AQGLQR.E	4.67/28667	0.15968	<	0.546563	3.42287	7.4033E-7	Cytoskeleton
4	71 Kd heat shock cognate protein	HSP7C	32,467	K.SFYPEEVSSMVLTK.M	5.37/71082	0.109243	<	0.256956	2.35214	0.000002	Protein translocation
5	14–3-3 protein epsilon isoform	YWHAE	902,787	R.VTIASLPR.N	4.63/29326	0.138728	<	0.408691	2.94598	0.000007	Signal transduction
6	Annexin A5	ANXA5	37,637	R.YLAEFATGNDR.K	4.94/35971	0.06177	<	0.188239	3.04744	0.000235	Signal transduction
7	Annexin A5	ANXA5	37,637	R.LYDAYELK.H	4.94/35971	0.116321	<	0.252109	2.16735	0.000010	Signal transduction
Group 2											
8	moesin, isoform CRA_b		119,625,804	K.GLGTDEESILTLLTSR.S	5.90 /66678	0.184177	<	0.700791	3.80498	0.000061	Cytoskeleton
Group 3											
9	Glucose-regulated protein 78 precursor, partial	GRP78	386,758	R.TWNDPSVQQDIK.F	5.03 /72185	0.803415	<	0.825214	1.02713	0.714026	Signal transduction
Group 4											
10	glyceraldehyde-3-phosphate dehydrogenase	GAPDH	31,645	R.GALQNIIPASTGAAK.A	8.26 /36202	0.560738	>	0.158456	0.28258	0.000761	Catalyzing reduction

### Cluster analysis

**Fig. 3:**
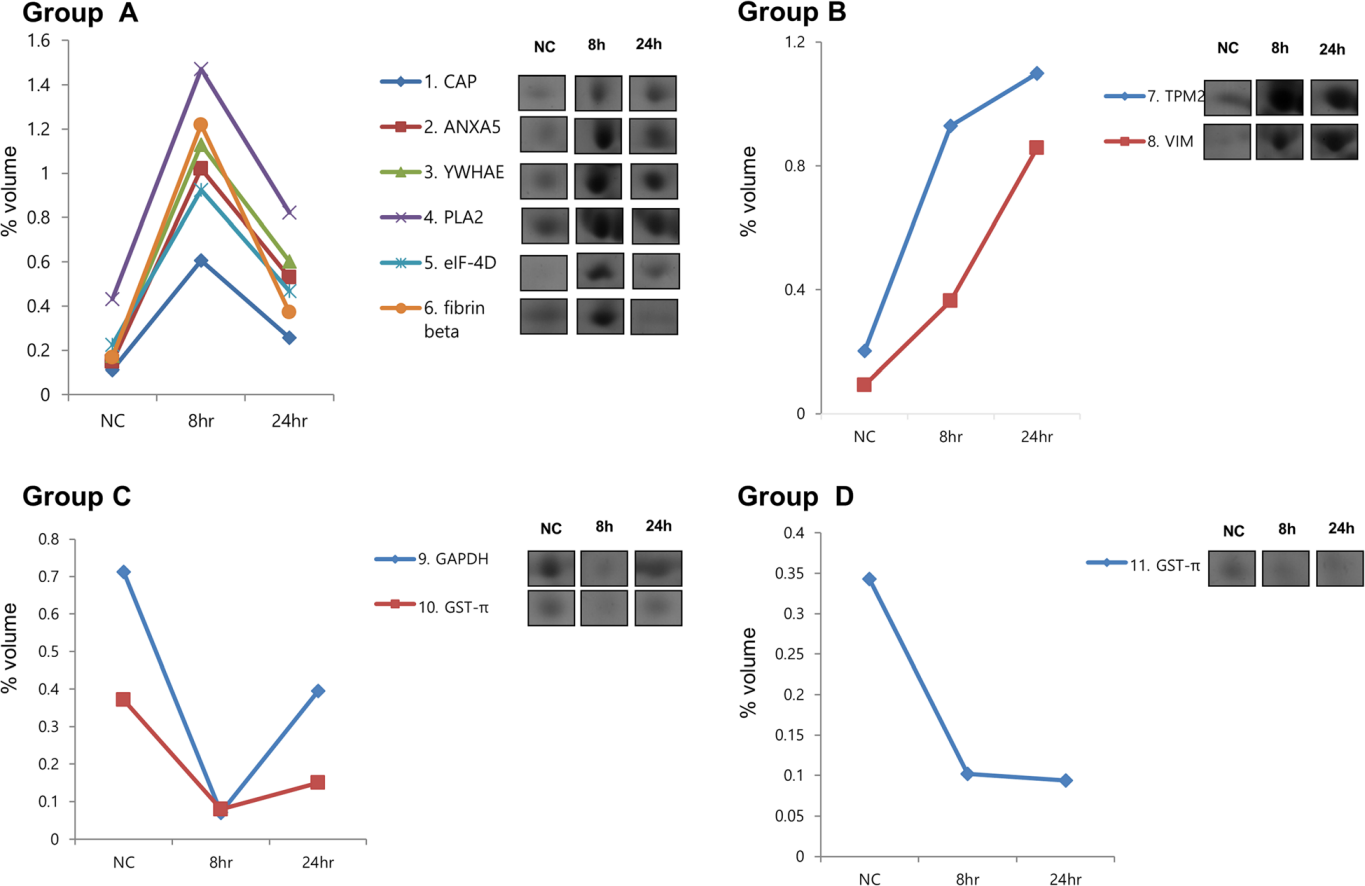
Cluster analysis of 11 proteins with significant differential expression (>3-fold change) at 8 or 24 h caused by 30 nm acrolein treatment of human lung microvascular endothelial cells. The expression profiles of the individual proteins were classfied by cluster analysis. Protein names National Center for Biotechnology Information (NCBI) are displayed for each cluster

**Fig. 4:**
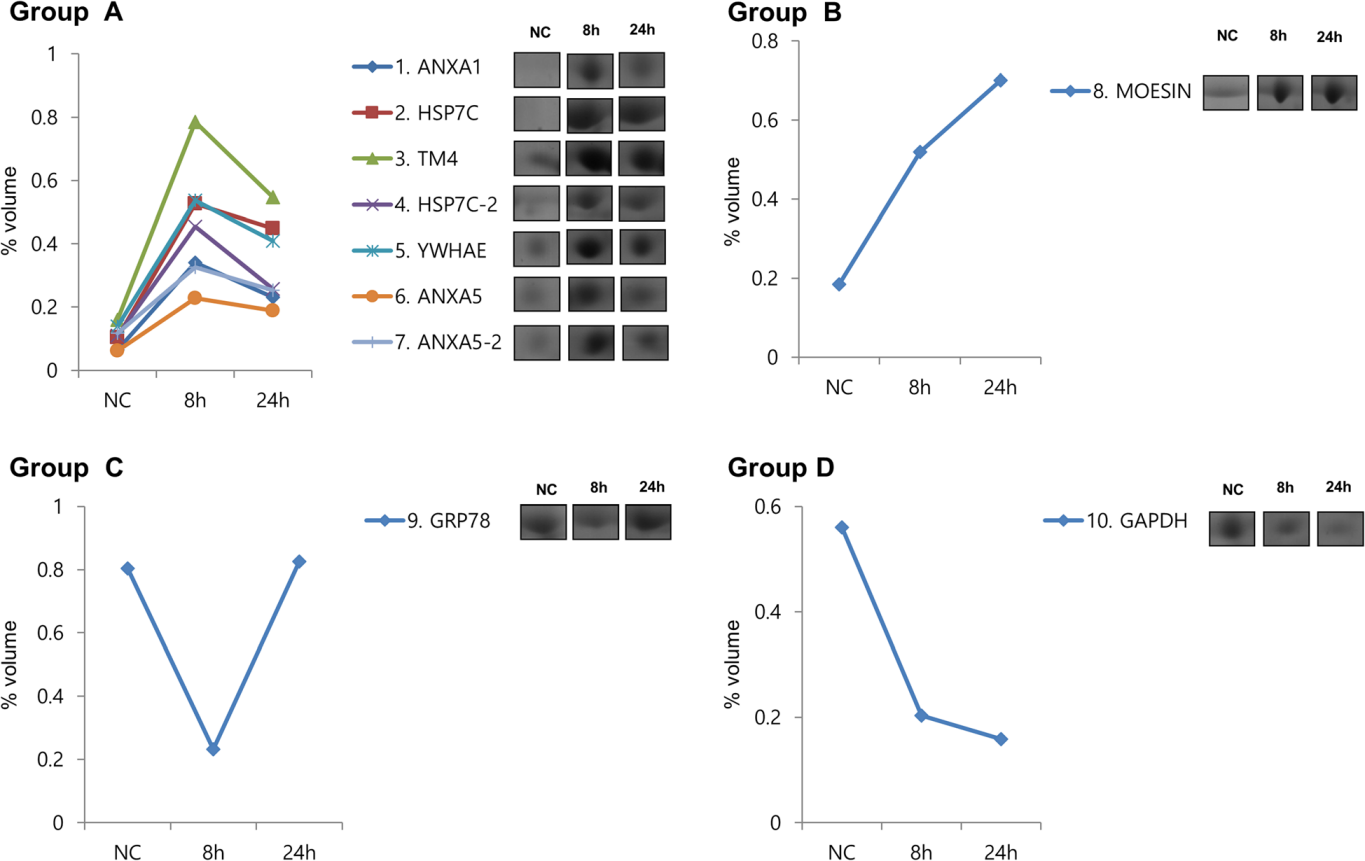
Cluster analysis of 10 proteins with significant differential expression (>3-fold change) at 8 or 24 h caused by 300 nm acrolein treatment of human lung microvascular endothelial cells. The expression profiles of the individual proteins were classfied by cluster analysis. Protein names National Center for Biotechnology Information (NCBI) are displayed for each cluster

The expression profiles of the 21 proteins with significant (*p <* 0.05) differential expression were visualized using a hierarchical clustering algorithm. Four fundamental profile patterns could be identified from the clusters: transiently increasing (Figs. 3 and 4, Group A), continuously increasing (Figs. 3 and 4, Group B), transiently decreasing (Figs. 3 and 4, Group C), and continuously decreasing (Figs. 3 and 4, Group 4).

Treatment of human lung microvascular endothelial cells (HMVEC-L) with acrolein 30 nM altered 11 protein spots on the two-dimensional gel, and these were then analyzed by MALDI-TOF MS. These proteins included antioxidant, signal transduction, cytoskeleton, protein transduction, catalytic reduction.

The proteins were classified into four groups according to the time course of their expression patterns such as continually increasing, transient increasing, transient decreasing, and continually decreasing. The proteins in each group are summarized in Table 1, 2, 3 and 4. The Adenylyl cyclase-associated protein (CAP), Annexin A5 (ANXA5), 14–3-3 protein epsilon isoform (YWHAE), phospholipase A2 (PLA2), initiation factor 4D (eIF-4D) and fibrin beta were included in group A. The Tropomyosin beta chain (TPM2) and Vimentin (VIM) were in group B.

A continuous decrease in Glutathione S-transferase P (GST-π) was observed in group C. A transient decrease in glyceraldehydes-3-phosphate dehydrogenase (GAPDH) and Glutathione S-transferase P (GST-π) was observed in group D.

Treatment of HMVEC-L with acrolein 300 nM altered 10 protein spots on the 2-D, and these were then analyzed by MALDI-TOF MS. These proteins included signal transduction, cytoskeleton, protein transduction, catalytic reduction. The proteins were classified into four groups according to the time course of their expression patterns such as continually increasing, transient increasing, transient decreasing, and continually decreasing. The Annexin A1 (ANXA1), 71Kd heat shock cognate protein (HSP7C), Tropomyosin 4 (TPM4), 71Kd heat shock cognate protein (HSP7C), 14–3-3 protein epsilon isoform (YWHAE), Annexin A5 (ANXA5) were included in group A. The Moesin, isoform CRA_b was in group B. A continuous decrease in glyceraldehydes-3-phosphate dehydrogenase (GAPDH) was observed in group C. A transient decrease in Glucose-regulated protein 78 precursor, partial (GRP78) was observed in group D.

### Identification of Moesin-expressing HMVEC-L in acrolein-treated mice lung

**Fig. 5:**
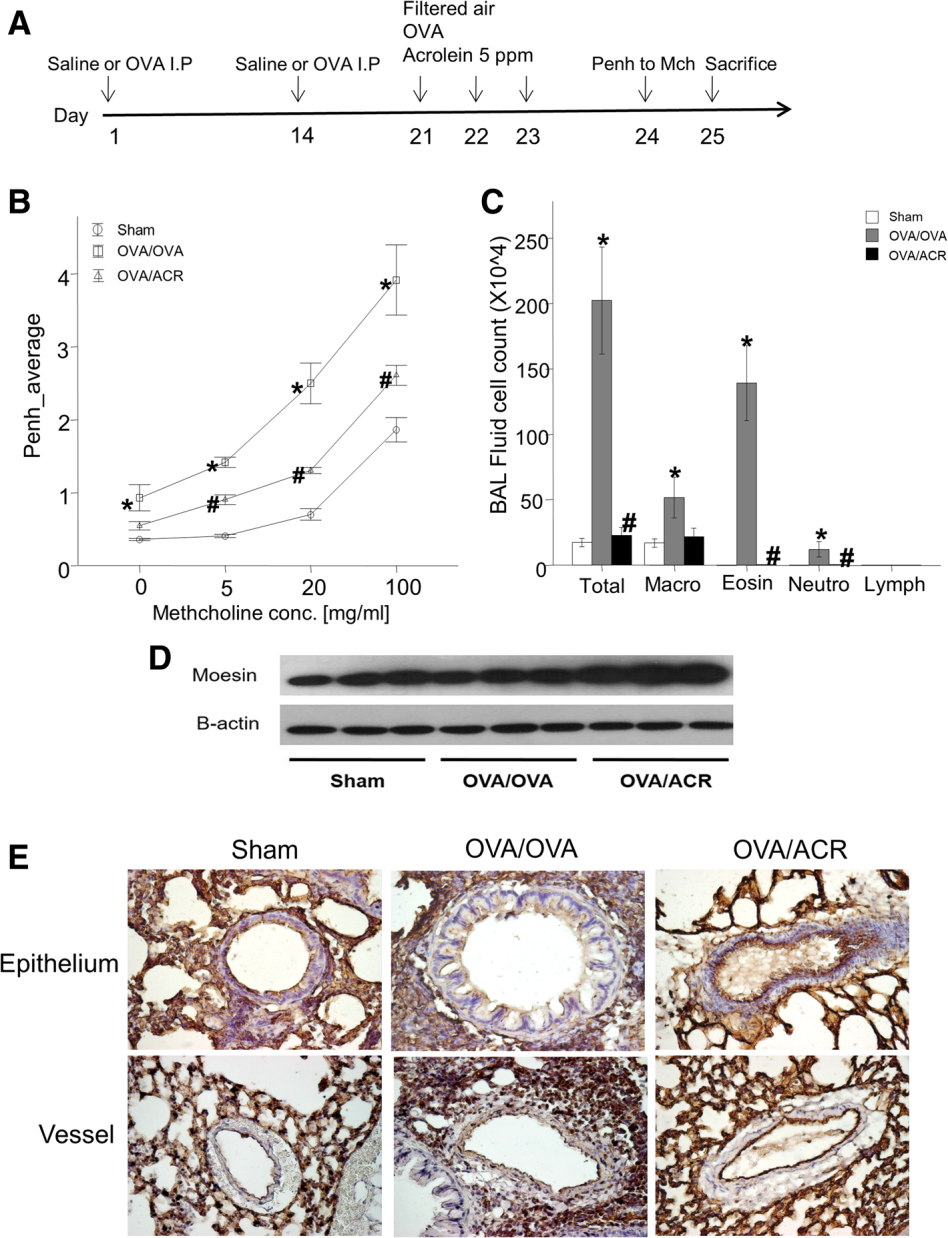
Experiment protocol of asthma model and expression of Moesin in lung tissue of acrolein-treated and sham-treated mice. **a**. OVA/OVA were sensitized intraperitoneal injection (I.P) at day 0, 14 to BALB/c. All mice received intranasal treatment (I.N) at day 21 to 23 with or without acrolein 1 h before OVA challenge. **b** and **c**. acrolein-treated mice increased cell differentials and airway hyperresponsiveness (AHR). **d**. Western blot analysis of lung cell extracts from sham- or acrolein-treated mice. Moesin expression in lung cells from OVA plus acrolein-treated mice was higher than in lung cells from sham-treated mice. **e**. lung tissues from acrolein-treated and sham-treated mice were incubated with biotinylated anti-goat Moesin antibody (1100 dilution). Moesin was detected using an avidin-biotin peroxidase complex kit and staining with 3,3′-diaminobenzidine tetrachloride (Zymed Laboratories Inc.) with hematoxylin as a counterstain. Moesin protein expression was notably higher in the lung endothelial cells and epithelial cells from acrolein-treated mice than in that from sham-treated mice

To determine whether Moesin and the other proteins expression in lung tissue is elevated after in vivo stimulation with acrolein, we exposed ovalbumin (3μg/ul) for 3 days or acrolein in mice for 5 ppm for 3 days. The acrolein-exposed mice group exhibited a greater increase in Penh compared to sham mice group (Fig. 5b). Examination of the BALF showed increased total and differential cell counts in the acrolein-exposed mice group compared to cell counts in the sham mice group (Fig. 5c). Expression of Moesin protein in intrapulmonary bronchi was examined by immunohistochemical staining. After acrolein 5 ppm exposure for 3 days, Western blot analysis of homogenates from sham- and acrolein-exposed mice lung lysates (20μg of protein/lane) showed that Moesin protein significantly increased in lung from acrolein- treated mice compared with that from untreated mice (Fig. 5d). Moesin was detected in the endothelial cell, epithelial cells of the bronchi and bronchioles, and inflammatory cells (Fig. 5e). In addition, Those results for the other two biological replicates revealed that the acrolein-exposed mice group and the acrolein-OVA exposed mice group significantly increased two proteins (Annexin A1 and Tropomyosin 2) compared to sham mice group (see Additional file 1).

## Discussion

Respiratory diseases are increasing cause of morbidity and mortality for all age groups and races in the changing global environment [30]. Despite differences in the causal agents, both asthma and chronic obstructive lung disease (COPD) exhibit various degrees of inflammatory changes, airway narrowing leading to airflow limitation and structural alterations of the pulmonary airways and vessels [31–33].

The involvement of residential cells such as endothelial cells, airway smooth muscle cells (ASM) and pulmonary fibroblasts, all appear to have a crucial role in the progression of vascular inflammation and remodeling [30, 34]. The density of vessels in the medium (2–5 mm inner diameter) and small (<2 mm) airways is significantly elevated in mild and moderate-to-severe asthma compared to healthy controls or subjects with COPD [32] and has been linked to asthma severity [35, 36]. Ongoing inflammatory processes in asthma may result in structural changes in the pulmonary vasculature and contribute to an influx of progenitors and increased synthesis of vascular mediators [30]. However, more studies are warranted to increase the knowledge of the cells and mediators associated with the vascular changes involved in the development of airway obstruction and remodeling processes in asthma [30].

Acrolein is a respiratory irritant that can be generated during cooking and is in environmental tobacco smoke [19, 37–40]. Acrolein is also generated endogenously at sites of injury, and excessive breath levels and induced sputum have been detected in asthma and COPD [19, 25, 41]. Because of its reactivity with respiratory-lining fluid or cellular macromolecules, acrolein alters gene regulation, inflammation, mucociliary transport, and alveolar–capillary barrier integrity [19].

Therefore in this study we have examined the effect of acrolein on human lung vascular endothelial cells and lungs of mouse model of asthma. Our data revealed four patterns of protein expression groups according to the time course of their expression patterns such as continually increasing, transient increasing, transient decreasing, and continually decreasing. And acrolein 30 nM altered 11 proteins including antioxidant, signal transduction, cytoskeleton, protein transduction, catalytic reduction such as CAP, ANXA5, YWHAE), PLA2, eIF-4D, fibrin beta, TPM2, VIM, GST-π, and GAPDH. Acrolein 300 nM altered 10 protein spots such as ANXA1, HSP7C, TPM4, YWHAE, ANXA5, Moesin, isoform CRA_b, GAPDH, and GRP78. Further studies will be needed for different expression pattern by different acrolein concentration.

Tropomyosin (TPM) is a component of the muscle sarcomeric thin filament where it plays a central role in the calcium-dependent regulation of striated muscle contraction. TPM exists as a rod-shaped dimer that forms a head-to-tail polymer along the length of an actin filament providing stability and it is essential for myosin-actin interaction [42–45]. Annexins are a family of 13 structurally related, Ca-dependent, phospholipid binding proteins [46] that modulate numerous physiological and pathological processes, including anticoagulation, anti-inflammation, cell migration, proliferation, apoptosis, and membrane-trafficking activities [47–50]. Ezrin/Radixin/Moesin (ERM) proteins are important actin-binding molecules that are targets for threonine phosphorylation in various signal pathways to induce actin remodelling and control vascular permeability [51, 52].

Of the 21 proteins we identified, we selected moesin for validation for airway inflammation in a mouse model of asthma. We found that moesin protein expression was increased in acrolein treated mice as compared with sham mice (Fig. 5). This result agrees with our results from 2-D electrophoresis, suggest that moesin can be an important role as a marker of airway inflammation. The immunopathogenesis of the acrolein model of airway inflammation has been considered to be quite different from that of the ovalbumin-challenged model. To our knowledge, our results provide the first clear demonstration that moesin in endothelial cells is induced by acrolein, suggesting that moesin have an important role for airway responsiveness and airway inflammation.

## Conclusion

The authors identified 21 proteins whose expression levels in the HMVEC-L cell line changed in response to acrolein exposure. These proteins include antioxidant, signal transduction, cytoskeleton, protein transduction, catalytic reduction implicated in the response to oxidative stress, and they can be classified into four groups according to the pattern of their acrolein-induced change in expression over time. One of these proteins, expression of moesin protein was increased in the lungs of acrolein-treated mice. These results indicate that some of these proteins may serve as mediators of, or markers for, airway disease caused by exposure to acrolein.

## Additional file

Additional file 1: Figure S1. A. Western blot analysis of lung protein extracts from sham- or acrolein-treated mice. Annexin A1 and Tropomyosin 2 expression in OVA plus acrolein-treated mice was higher than in sham-treated mice. B. lung tissues from acrolein-treated and sham-treated mice were incubated with biotinylated anti-rabbit Annexin A1 and Tropomyosin 2 antibody (1:500 dilution). Annexin A1 and Tropomyosin was detected using an avidin-biotin peroxidase complex kit and staining with 3,3′-diaminobenzidine tetrachloride (Zymed Laboratories Inc.) with hematoxylin as a counterstain. Annexin A1 and Tropomyosin 2 protein expression was higher in acrolein-treated mice than in that from sham-treated mice. (DOCX 1404 kb)

## Abbreviations

2-DE: Two-dimensional electrophoresis
ACN: Acetonitrile
BALF: Bronchoalveolar lavage fluid
COPD: Chronic Obstructive Pulmonary Disease
HMVEC-L: Human Microvascular Endothelial Cells-Lung
MALDI-TOF MS: Matrix-assisted laser desorption/ionization time-of-flight mass spectrometry
