# Supplementary material for: Proteomic identification of moesin upon exposure to acrolein
Source: Proteome Sci. 2018 Jan 17;16:2. doi: 10.1186/s12953-017-0130-4 (PMC5773073; doi:10.1186/s12953-017-0130-4)

Figure S1. A. Western blot analysis of lung protein extracts from sham- or acrolein-treated mice. Annexin A1 and Tropomyosin 2 expression in OVA plus acrolein-treated mice was higher than in sham-treated mice. B. lung tissues from acrolein-treated and sham-treated mice were incubated with biotinylated anti-rabbit Annexin A1 and Tropomyosin 2 antibody (1:500 dilution). Annexin A1 and Tropomyosin was detected using an avidin-biotin peroxidase complex kit and staining with 3,3‘-diaminobenzidine tetrachloride (Zymed Laboratories Inc.) with hematoxylin as a counterstain. Annexin A1 and Tropomyosin 2 protein expression was higher in acrolein-treated mice than in that from sham-treated mice.


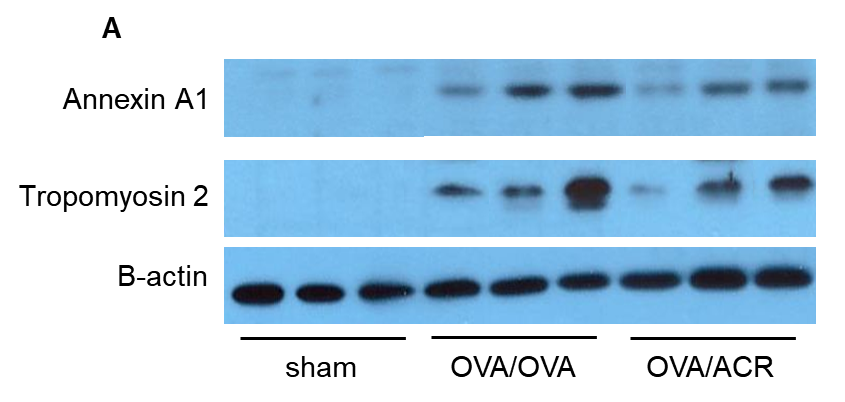


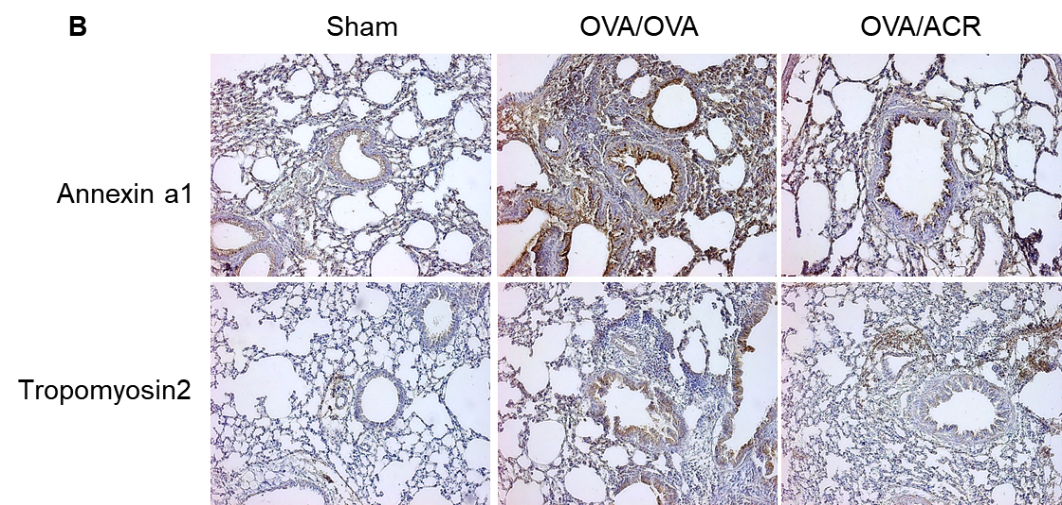

Supplement: Supplementary file 1 — A. Western blot analysis of lung protein extracts from sham- or acrolein-treated mice. Annexin A1 and Tropomyosin 2 expression in OVA plus acrolein-treated mice was higher than in sham-treated mice. B. lung tissues from acrolein-treated and sham-treated mice were incubated with biotinylated anti-rabbit Annexin A1 and Tropomyosin 2 antibody (1:500 dilution). Annexin A1 and Tropomyosin was detected using an avidin-biotin peroxidase complex kit and staining with 3,3′-diaminobenzidine tetrachloride (Zymed Laboratories Inc.) with hematoxylin as a counterstain. Annexin A1 and Tropomyosin 2 protein expression was higher in acrolein-treated mice than in that from sham-treated mice. (DOCX 1404 kb) [file 12953_2017_130_MOESM1_ESM.docx]
